# Evaluation of colorectal cancer liver metastases based on liquid biopsy combined with folate receptor– Positive circulating tumor cells and HSP90

**DOI:** 10.3389/fonc.2022.912016

**Published:** 2022-09-20

**Authors:** Maosen Huang, Linyao Cheng, SiSi Mo, Haiming Ru, Xianwei Mo, Linhai Yan

**Affiliations:** ^1^ Department of Gastrointestinal Surgery, Guangxi Medical University Cancer Hospital, Nanning, China; ^2^ Department of Gastrointestinal Surgery, Guangxi Clinical Research Center for Colorectal Cancer, Nanning, China; ^3^ Department of Gastrointestinal Surgery, Guangxi Key Laboratory of colorectal cancer prevention and Treatment, Nanning, China

**Keywords:** FR+CTC, HSP90, Liquid biopsy, LMCRC, CRC

## Abstract

**Objective:**

Liver metastasis of colorectal cancer (LMCRC) is a major cause of cancer-related deaths worldwide. We can reduce the mortality rate by discerning the risk of liver metastases in patients with colorectal cancer at an early stage. Hence, we combined the use of folate receptor (FR)–labeled circulating tumor cells (FR+CTCs) and the metastasis-related marker, heat shock protein 90 (HSP90), to screen patients with colorectal cancer and explore the prognostic factors of patients with high expression of FR+CTC and HSP90.

**Patients and methods:**

A retrospective study of 356 patients with measurable colorectal cancer was performed. Negative enrichment and FR-targeted fluorescence quantitative PCR was utilized to detect FR+CTC. An ELISA kit was used to detect HSP90 expression. A timely follow-up study of patients with colorectal cancer was made.

**Results:**

Colorectal patients with liver metastases showed high expression of FR+CTCs and HSP90. The diagnostic ability of the combined receiver operating characteristic curve of FR+CTC and HSP90 (area under the curve [AUC]=0.79, sensitivity 70.55%, specificity 92.66%) was significantly greater than that of a single index. The results of timely follow-up of patients showed that the high expression of FR+CTC significantly shortened the median disease-free survival (mDFS) of 36.5 months (95% confidence interval [CI]: 14.13–58.87, Logrank *p* < 0.0001) compared with the low expression cohort. The mDFS of the HSP90 high-expression cohort was significantly higher than that of the low-expression cohort (Logrank *p* = 0.0002), mDFS=58.47 months (95% CI: 37.12–79.81, Logrank *p* < 0.0001). We performed univariate and multivariate analyses to show that FR+CTC and HSP90 were risk factors for the progression of metastatic colorectal cancer (MCRC) disease. We then constructed a high- and low-risk score model of risk factors to evaluate MCRC. The diagnostic sensitivity of the risk model for MCRC was significantly improved (AUC=0.89, sensitivity 85.29%, specificity 81.33%), and the mDFS of patients in a high-risk group increased to 33.28 months (95% CI: 27.24–39.31, Logrank *p* < 0.0001). The establishment of the model improves the early screening of patients with MCRC.

**Conclusion:**

Patients with colorectal cancer and high expression of FR+CTC and HSP90 are at risk of liver metastasis and this suggests a poor prognosis. Combining the two markers can improve the early screening and diagnosis of LMCRC patients. In addition, combining a multivariate risk model can further assist patients in appropriate stratification and the design of tailored treatment regimens. However, further validation these markers is needed before their routine clinical application.

## Introduction

Colorectal cancer ranks third in incidence and second in mortality of all cancers worldwide, accounting for approximately 10% of cancers and cancer-related deaths, respectively (1). Less than 20% of patients survive for more than 5 years. Metastatic colorectal cancer (MCRC) is one of the most important factors affecting mortality. The probability of metastasis in stage III patients is as high as 50%, and the liver is the most common site of distant metastasis (2). In recent years, it has been shown that individualized treatment of molecular and pathological characteristics of tumors can improve overall survival. For first-line and second-line chemotherapy combined with bevacizumab, cetuximab, *BRAF* and epidermal growth factor receptor (EGFR) inhibitors, and immunotherapy, it can effectively prolong the median survival of patients with advanced cancer. However, the highly heterogeneous nature of colorectal cancer poses enormous difficulties in treatment. There is currently no effective targeted therapy for 35% to 40% of colorectal cancer patients (3). Another problem affecting the prognosis of metastatic colorectal cancer is that few biomarkers currently known that show extreme sensitivity, high specificity, and convenience for the direct monitoring of liver metastasis. It is also difficult to obtain sequential tumor tissues for analysis. Exploring new markers can reveal an early metastatic response or treatment effect, and contribute to the precise diagnosis and treatment of metastatic colorectal cancer, which is crucial for the early detection of metastasis and exploration of personalized treatment.

In recent years, the term “liquid biopsy” has gradually been integrated into the detection of colorectal cancer: Circulating tumor cells (CTCs) can enter the circulatory system of patients with tumors and be detected in the peripheral blood, which is closely linked to tumor metastasis. Such cells have been used to monitor the continuity of prognosis and drug efficacy in patients with colorectal cancer (4). This includes a CellSearch^®^ system to detect antibodies for epithelial cell adhesion molecule (EpCAM), and the immunomagnetic enrichment of ferrofluids. However, the absence of certain tumor epithelial cell–specific markers, and the use of biophysical isolation methods lack specificity, allowing superposition and confusion between CTCs and leukocytes (5). Therefore, the current sensitivity and specificity of CTC detection is low. Folate receptor (FR) is a glycosylphosphatidylinositol-anchored protein with high affinity for folic acid and N5-methyltetrahydrofolate. Folate receptor α is the most commonly expressed isoform and is involved in intracellular and extracellular folate transport. Moreover, FR is currently highly expressed in colorectal cancer epithelial cells, but is expressed at a lower level in normal tissue epithelial cells, except for macrophages (6). Studies have shown that FR positivity is independently associated with survival after hepatectomy for colorectal cancer and has a certain diagnostic value (7). Folate receptor has become a molecular target for the development of many cancer treatments, including biomarkers, PECT-marked imaging agents, and folate-conjugated drugs and toxins (8, 9). Therefore, FR can be invoked as a highly sensitive biomarker for identifying CTCs in the peripheral blood of patients with colorectal cancer.

Heat shock protein 90 (HSP90), as an influential member of a family of molecular chaperones, can easily induce the expression of HSP90 under stress. Heat shock protein 90 α is the most common isoform in eukaryotic cells (10). Many oncoproteins that promote tumor cell proliferation and metastasis are clients of HSP90. Unstable oncogenic mutations may increase the dependence of tumor cells on HSP90, including driver mutations such as *EGFR*, *BRAF*, and *AKT*, among others (11). Consequently, HSP90 levels are significantly elevated in tumor cells at multiple mutation sites (12). It has been reported that HSP90 related tumor migration (13). Targeting HSP90 can effectively inhibit the growth and liver metastasis of colorectal cancer and improve the efficacy of chemotherapy (14), demonstrating that HSP90 affects the process of liver metastasis of colorectal cancer cells. At present, chemotherapy, targeted therapy, and immunotherapy combined with HSP90 inhibitors have achieved certain curative effects in patients with advanced colorectal cancer (15).

We retrospectively collected data from 356 patients with colorectal cancer and divided the latter into metastatic and non-metastatic groups. In our study, we found that FR+CTC and HSP90 were highly expressed in patients with liver metastases and showed a certain correlation. Combining two metastasis-related markers can sharply distinguish liver metastasis and non-liver metastasis groups, and establish a risk score model for the early monitoring of the risk of liver metastasis in patients with colorectal cancer. Doctors should be promptly reminded to instigate clinical interventions for early diagnosis in order to improve the survival of patients with liver metastases and reduce mortality.

## Patients and methods

### Study design

This study is a retrospective study. From January 2017 to December 2021, a total of 356 patients with diagnosed colorectal cancer were included, and we strictly screened patients at their first visit. The inclusion criteria were: 1) first-diagnosed patients; 2) colorectal cancer was confirmed by pathological biopsy using a colorectal endoscope; and 3) computed tomography or PECT of colorectal cancer patients with liver metastases clearly showed liver metastases. The exclusion criteria were: 1) postoperative patients; 2) patients after chemotherapy, targeted therapy, and immunotherapy; 3) colorectal cancer metastases to other organs except liver metastasis; and 4) metastases from other cancers to colon or rectum. In addition, we excluded patients who were long-term vegetarians, or patients who required long-term dependence on folic acid drugs. In the end, 259 patients with non-liver metastatic colorectal cancer and 97 patients with liver metastatic colorectal cancer were enrolled in the study cohort. Three mL of venous blood was taken for FR+CTC and HSP90 detection at the patients’ initial visit. In addition, we followed up 259 colorectal patients with non-liver metastases in a timely manner. When the patients achieved clinical remission after standard surgery and first-line standard chemotherapy (Considering that each patient’s choice of chemotherapy regimen is different, we try our best to follow the NCCN guidelines for chemotherapy regimen selection, so that patients can achieve complete response as much as possible). We not only explored the serological indicators of FR+CTC in colorectal cancer patients (HSP90, CEA and CA199), but also explored the FR+CTC and related tumor tissue immunohistochemical indicators (P53, KI67, CK7, CK20, Villin, β-catenin, and S100), also explored the correlation of folic acid with tumor tissue size and the number of lymph node metastases. Better explain the biological behavior of colorectal cancer patients through the circulation of fluids from the primary tumor to distant metastases. The follow-up endpoint was the occurrence of liver metastases during the follow-up period. Follow up every three months, the most important thing is to follow up the patient’s imaging examination (CT or PECT) for a long time, if there are signs of liver metastases or liver hypermetabolism lesions, the radiologist Diagnosed as liver metastases (**Supplementary Figure 1**). The treatment of all patients included in this study, as well as sample collection and research, were conducted in accordance with the regulations promulgated by the National Health Commission of China and the ethics standards established in the Declaration of Helsinki. Written informed consent was given by all patients. Retrospective study permission was granted by the Institutional Review Board of Guangxi Medical University Cancer Hospital (KY2021279)

### FR+CTC analysis

A CytoploRare^®^ Circulating CRC cell kit was provided by GenoSaber Biotech Co., Ltd. (Shanghai, China). The kit consisted of two components: one is for CTC enrichment and the other is for CTC detection and quantification. The enrichment component included red cell lysis buffer, incubation buffer, anti-CD45 leukocyte depletion magnetic beads, washing buffer, labeling buffer, stripping buffer, and neutralization buffer. The detection and quantification component included PCR reaction buffer, primers, deionized water, positive and negative cell controls, PCR controls, and standards. The primer sequences were listed as follows: RT primer, 59-CTCAACTGGTGTCGTGGAGTCGGCAATTCAGTTGAGGGTTCTAA-39; Forward primer, 59-TATGATTATGAGGCATGA-39; Reverse primer, 59-GGTGTCGTGGAGTCG-39; Taqman Probe, 59-FAM-CAGTTGAGGGTTC-MGB-39. Following the manufacturer’s instruction manual, CTCs were enriched by lysis of erythrocytes and immunomagnetic depletion of leukocytes from 3 mL blood samples. Enriched CTCs were labeled with a conjugate of a tumor-specific ligand, folic acid, and a synthesized oligonucleotide. After labeling, enriched CTCs were washed thoroughly to remove the unbound conjugates. Subsequently, the bound conjugates were specifically stripped from the CTC surface and collected for quantitative PCR analysis. Before amplification, the conjugate first annealed and extended on the RT primer. After that the extended conjugate was amplified and analyzed using a Taqman probe–based quantitative PCR method. In the above method, circulating tumor cells were identified as FR-positive cells after labeling with folate-linked oligonucleotides.

### HSP90 analysis

Plasma HSP90 levels were detected by HSP90 protein ELISA kit (Antai Protegen Biotechnology Development Co., Ltd., Antai, China). Fresh blood samples (3 mL) were harvested from patients and controls in combination with EDTA-K2 anticoagulant. All steps were performed according to the manufacturer’s instructions. Fresh blood samples were first pre-incubated at 37°C for 30 min, then centrifuged at 3000 rpm for 10 min and diluted 20-fold with the provided diluent. Prepared samples (50 µL each) were added to a 96-well plate, followed by 50 µL of anti-HSP90 horse radish peroxidase-labeled antibody. These were incubated at 37°C and the samples were gently shaken for 1 h. Next, the plate was washed six times with the wash buffer provided in the kit, and then the color reaction was performed: 50 µL of peroxide and 50 µL of 3, 3, 5, and 50 µL of tetramethylbenzidine were added, respectively, at 37°C and incubated for 20 min. The reaction was terminated by the addition of acid stop buffer. Finally, the optical density was measured by spectrophotometer with an excitation wavelength of 450 nm and a detection wavelength of 620 nm as a reference wavelength. The concentration of HSP90 protein in each sample was calculated according to a standard curve of optical density values.

### Statistical analysis

The difference between two groups was tested by a nonparametric test, and *P* < 0.05 was considered statistically significant. The two groups of data were continuous numerical variable data, and the mean ± standard error of the mean (SEM) was used for a statistical description. A receiver operating characteristic (ROC) curve was utilized to explore the diagnostic ability of relevant markers in patients with colorectal cancer and liver metastasis and those without liver metastasis. Its diagnostic ability was determined by the area under the curve (AUC). Cutoff values ​​were calculated on the basis of the Youden index (sensitivity + specificity – 100%). In this manner, the sensitivity and specificity of comparable cut-off values were known. Follow-up data were collected from patients included in the study, and median disease-free survival (mDFS) was assessed using a stratified Logrank test. Through univariate and multivariate logistic regression analyses, the prognostic factors affecting the prediction of colorectal cancer liver metastasis were explored. Based on research data and after screening meaningful variables, a high- and low-risk scoring model was established. According to a multivariate risk regression model, we obtained the expression coefficient of each independent risk gene. Taking into account this risk factor, we established a DFS prediction model for predicting the risk of liver metastases and standardized the expression of risk factors by a zero-centered method to establish a risk-scoring model for clinical reference. Data were analyzed in this study by SPSS software (SPSS 17.0, Chicago, Illinois, USA) and Graphpad Prism Version 7.0 software (La Jolla, CA, USA).

## Results

### Clinical characteristics of liver and non-liver metastasis cohorts at baseline

**Figure 1:**
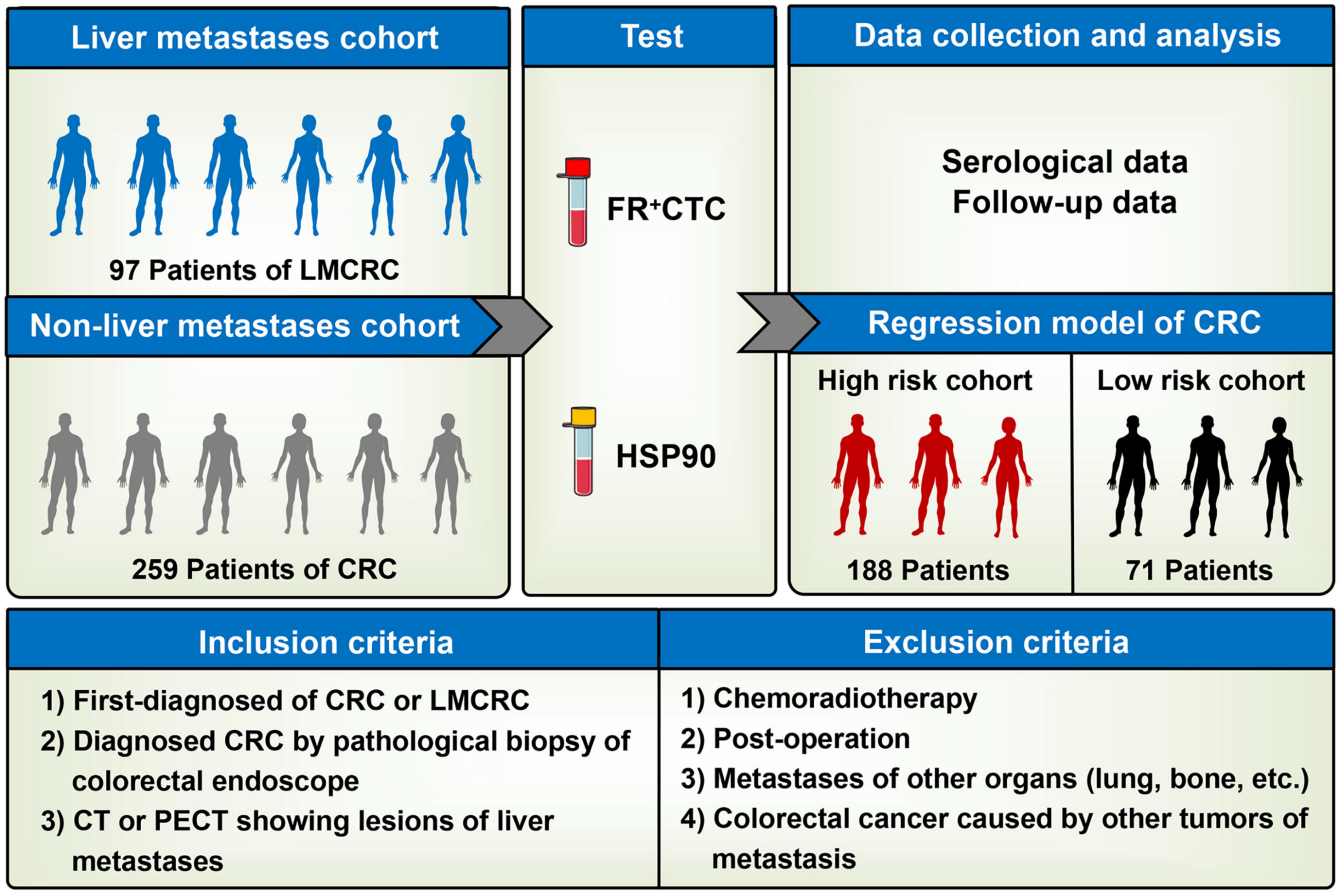
Flow chart of the study: The cohort in this study consisted of colorectal cancer patients with and without liver metastasis.

A total of 450 participants were recruited from January 1, 2017 to December 31, 2022. After screening according to the inclusion and exclusion criteria, 259 patients with without liver metastatic colorectal cancer and 97 patients with liver metastatic colorectal cancer were selected (without overlapping patients in the two cohorts). Next, 2–3 mL of venous blood for FR+CTC and HSP90 was taken from 356 patients with colorectal cancer that were incorporated into the study cohort. Clinical data was prospectively collected, including serological data (such as tumor markers, immunohistochemical indexes, maximum diameter of tumor, number of lymph node metastases) and clinical follow-up data. Relevant clinical markers were statistically analyzed to explore whether FR+CTC and HSP90 could be used to monitor the risk of liver metastasis and recurrence in patients with colorectal cancer. Finally, risk factors related to FR+CTC and HSP90 were screened according to univariate and multivariate logistic regression analyses. When a cumulative regression equation was obtained, the risk score was calculated to predict the risk of liver metastasis in patients with colorectal cancer. A high-risk model cohort was further constructed to guide the clinical risk assessment. The model, composed of a high-risk cohort of 159 patients and low-risk cohort of 197 patients, was capable of early predictions of the risk of liver metastasis from colorectal cancer, with timely intervention to prevent and reduce the recurrence rate and mortality (**Figure 1**).

**Figure 2:**
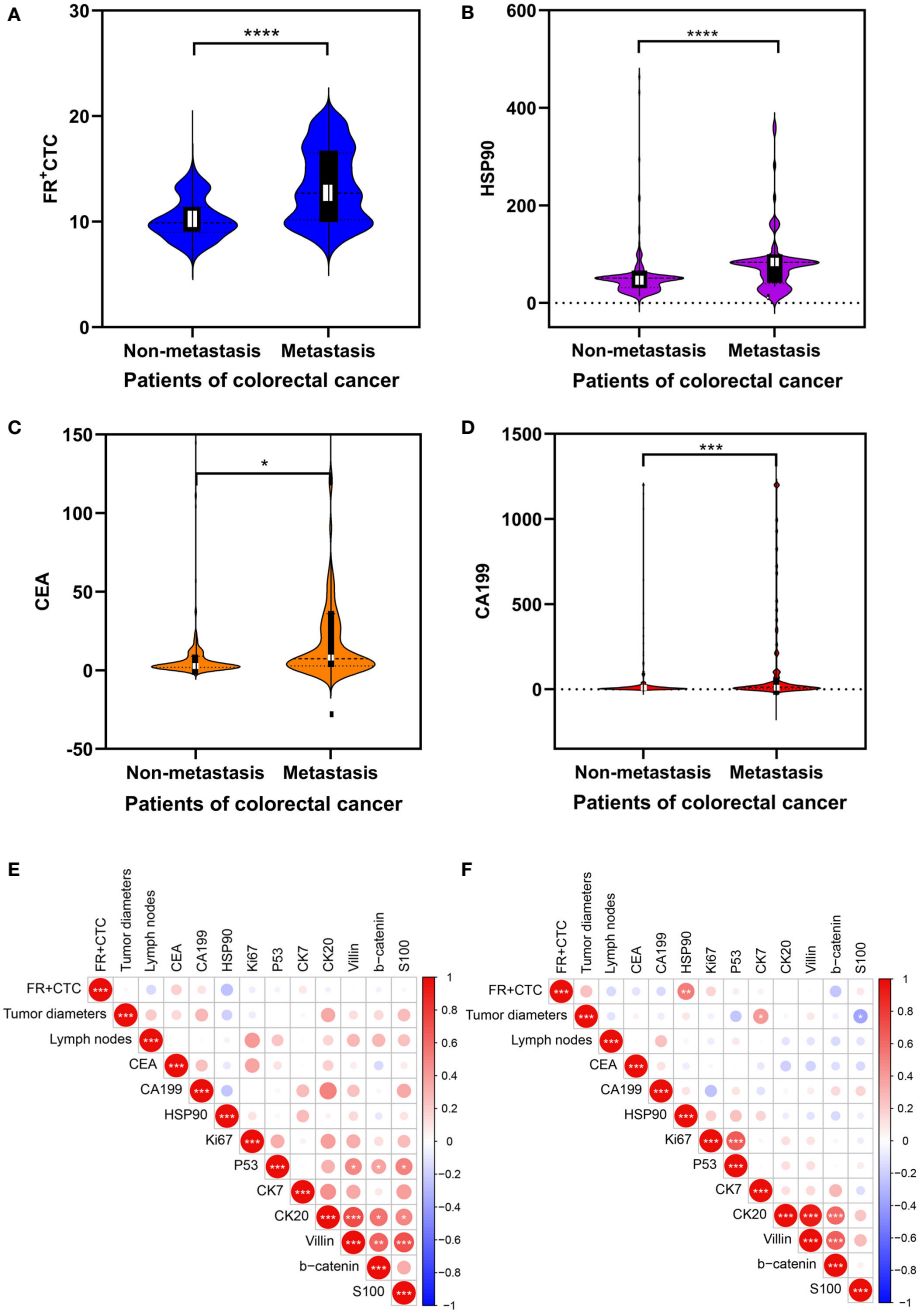
Analysis of clinical data of cohorts: **(A–D)** Comparison of the serology of folate receptor plus circulating tumor cells (FR+CTC), heat shock protein 90 (HSP90), CEA, and CA199 levels between colorectal cancer patients with and without liver metastasis. The data are presented as mean ± standard error of the mean (SEM). Heatmap expression matrix of serological marker correlations in non-metastatic **(E)** and metastatic colorectal cancers **(F)**. “*” Represents the p-value of the two groups after statistical test, * represents p<0.05, ** represents p<0.01, *** represents p<0.001, and **** represents p<0.0001.

Demographic characteristics, FR+CTC, HSP90, and other detection indices of non-liver and liver metastatic colorectal cancer cohorts are presented in **Table 1**. The age and gender of the two groups were compared. Significant differences were found in FR+CTC (*P* < 0.0001, Wilcoxon test) and HSP90 (*P* < 0.0001, Wilcoxon test) between the two groups (**Figures 2A, B**). The average levels of FR + CTC and HSP90 in the non-liver metastatic group were 10.26 ± 0.12 and 51.16 ± 2.87. While in the liver metastatic group, the average levels of FR+CTC and HSP90 were 13.38 ± 0.35 and 78.19 ± 6.10 respectively. The average levels of FR+CTC and HSP90 in the blood of the liver metastasis cohort were markedly increased. This result was consistent with the trend we expected. These markers had considerable potential in predicting liver metastasis of colorectal cancer. Compared with conventional colorectal cancer–related markers, such as CEA and CA199, the significant difference between non-liver and liver metastasis cohorts was less than that of FR+CTC, HSP90 (**Figures 2C, D**), CEA (*P* < 0.05, Wilcoxon test), and CA199 (*P* < 0.01, Wilcoxon test). Moreover, compared with CEA and CA199, FR+CTC and HSP90 have a smaller range of 95% CI. In brief, to a certain extent, FR+CTC and HSP90 show better stability in predicting liver metastasis in patients with colorectal cancer.

**Table 1:** Comparisons of parameters between patients with colorectal cancer and liver and non-liver metastases.

Parameter	Non-Liver metastases N= 259 (means ± SEM) or (%)	Liver metastases N= 97 (means ± SEM) or (%)
**Age**	58.38 ± 0.79	56.93 ± 1.17
**FR-CTC**	10.26 ± 0.12 ****	13.38 ± 0.35 ****
**HSP90**	51.16 ± 2.87 ****	78.19 ± 6.10 ****
**CEA**	39.79 ± 9.69 *	84.59 ± 26.70 *
**CA199**	41.09 ± 9.55 ***	126.10 ± 28.33 ***
**KI67**	69.81 ± 0.85	72.90 ± 1.39
**P53**	55.86 ± 1.16	52.21 ± 2.45
**Tumor diameter**	4.37 ± 0.11	4.80 ± 0.19
**Number of lymph node metastases**	1 ± 0.16 ****	3± 0.42****
**Gender **		
Male	104 (40.15)	58 (59.79)
Female	155 (59.85)	39 (40.21)
**CK7**		
Positive	15 (5.79)	8 (8.25)
Negative	244 (94.21)	89 (91.75)
**CK20**		
Positive	86 (33.20)	28 (28.87)
Negative	173 (66.80)	69 (71.13)
**Villin**		
Positive	70 (27.03)	27 (27.84)
Negative	189 (72.97)	70 (72.16)
**β-catenin**		
Positive	63 (24.32)	14 (14.43)
Negative	196 (75.68)	83 (85.57)
**S100**		
Positive	55 (21.24)	16 (16.50)
Negative	204 (78.76)	81 (83.50)
**Location of primary tumor**		
Colon	123(47.49)	56 (57.73)
Rectal	136 (52.51)	41 (42.27)
**Differentiation type**		
Well differentiation	18 (6.95)	3 (3.09)
Moderately differentiation	233(89.96)	88 (90.72)
Poorly differentiation	8 (3.09)	6 (6.19)

*p > 0.05 between liver metastases and non-liver metastases.

***p > 0.001 between liver metastases and non-liver metastases.

****p > 0.0001 between liver metastases and non-liver metastases.

We further analyzed the correlation of FR+CTC, HSP90, maximum diameter of the tumor, the number of lymph node metastases, and immunohistochemical markers of mutation, proliferation, and metastasis in the two cohorts, and visualized their correlation expression matrix. In the cohort of patients with non-liver metastasis, a significant correlation was not found between the expression of serum FR+CTC and HSP90, and various clinical indexes (*P* < 0.05, Spearman test; **Figure 2E**). In contrast, in the liver metastasis cohort, we found that FR+CTC was positively correlated with HSP90 (*P* < 0.01, Spearman test), while FR+CTC and HSP90 did not significantly correlate with other markers (**Figure 2F**). However, it can be observed that they show a certain correlation in regulating the liver metastasis of colorectal cancer. From the above preliminary discussion, we can speculate that the high expression of FR+CTC and HSP90 in patients’ sera can collectively influence liver metastasis to a certain extent.

### Receiver operating characteristic curves of FR+CTC and HSP90 improve the diagnostic efficiency of liver metastasis in patients with colorectal cancer

**Figure 3:**
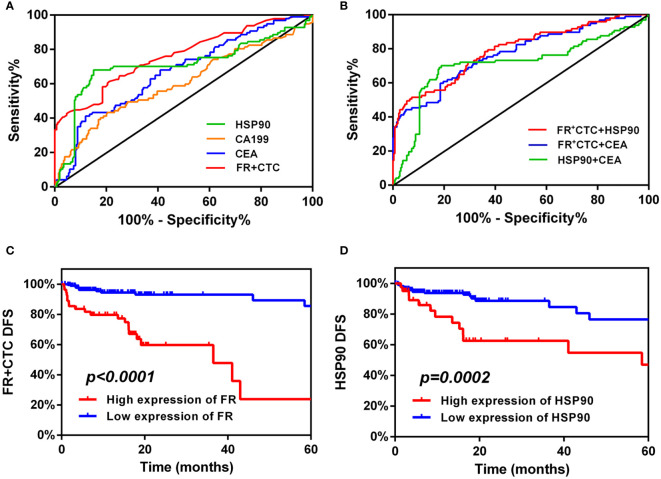
Diagnostic and prognostic capabilities of folate receptor plus circulating tumor cells (FR+CTC) and heat shock protein 90 (HSP90). **(A)** Receiver operating characteristic (ROC) curve analysis: the diagnostic ability of single FR+CTC, HSP90, CEA, and CA199 in differentiating liver metastatic colorectal cancer. **(B)** ROC curve analysis: the diagnostic ability of combined markers in differentiating liver metastatic colorectal cancer were FR+CTC and HSP90, FR+CTC and CEA, and HSP90 and CEA, respectively. High expression of FR+CTC **(C)** and HSP90 **(D)** predicts the median disease-free survival (mDFS) of liver metastatic colorectal cancer.

Based on the above exploration, we used a ROC curve to forecast the diagnostic efficiency of FR+CTC and HSP90 in the sera of 259 patients with non-liver metastatic colorectal cancer and 97 patients with liver metastatic colorectal cancer. We also compared common clinical markers, such as single CEA and CA199, between the two groups (**Figure 3A**). The AUC of CEA and CA199 was 0.67 (95% CI: 0.60–0.73) and 0.60 (95% CI 0.53–0.68), respectively (**Table 2**). However, the cut-off value of FR+CTC was 11.75, AUC: 0.6 (95% CI: 0.70–0.82), sensitivity was 61.46%, specificity was 78.76%; the HSP90 cut-off value was 55.65, AUC: 0.71 (95% CI: 0.64–0.78), sensitivity was 68.04%, and specificity was 84.94%. Compared with the traditional serological tumor markers, CEA and CA199, FR+CTC and HSP90 showed higher sensitivity and were better able to distinguish whether patients with colorectal cancer had liver metastasis.

**Table 2:** Diagnostic value of alone and combined biomarkers for distinguishing patients with colorectal cancer liver metastases (LMCRC).

Variables	AUC( Area under curve)	*p* value	Cut-off	Sensitivity	Specificity	Variables	
						Upper limit	Lower limit
**FR^+^CTC**	0.76	****	11.75	61.46	78.76	0.70	0.82
**HSP90**	0.71	****	55.65	68.04	84.94	0.64	0.78
**CEA**	0.67	****	17.04	41.24	88.03	0.60	0.73
**CA199**	0.60	****	18.95	43.30	79.54	0.53	0.68
**FR-CTC + HSP90**	0.79	****	–	70.55	92.66	0.73	0.84
**FR-CTC + CEA**	0.77	****	–	59.79	81.47	0.71	0.83
**HSP90 + CEA**	0.71	****	–	70.10	81.08	0.64	0.78

*p > 0.05 between liver metastases and non-liver metastases.

**p > 0.01 between liver metastases and non-liver metastases.

***p > 0.001 between liver metastases and non-liver metastases.

****p > 0.0001 between liver metastases and non-liver metastases.

It is worth noting that we further analyzed FR+CTC and HSP90, FR+CTC and CEA, and HSP90 and CEA (**Figure 3B**). As shown in **Table 2**, the AUC of the combined detection of FR+CTC and HSP90 was 0.79 (95% CI: 0.73–0.84), the sensitivity was 70.55%, and the specificity was 92.66%. The AUC, and diagnostic sensitivity and specificity were significantly higher than those of the combined detection of FR+CTC and CEA (AUC=0.77, 95% CI: 0.71–0.83), and HSP90 and CEA (AUC=0.71, 95% CI: 0.64–0.78). Thus, the combined detection of FR+CTC and HSP90 was better than the simple detection of FR+CTC or HSP90 in the diagnosis of liver metastasis in patients with colorectal cancer. The combined diagnosis greatly improved the detection efficiency. Therefore, the above results provide us with strong evidence that the combination of FR+CTC and HSP90 has certain advantages in predicting colorectal cancer liver metastasis, which is conducive to finding liver metastasis early and taking clinical intervention measures in time.

### FR+CTC and HSP90 can be used to evaluate the time from clinical remission (after initial treatment) to liver metastasis in patients with colorectal cancer

In a matched list of non-liver metastases, the patients with colorectal cancer included in our study achieved clinical remission after initial treatment. Our study continued to follow disease progression in 259 patients with non-liver metastases. The progression standard was whether liver metastasis was present, and the recurrence of liver metastasis, as suggested by pathology, CT or PECT, was included in follow-up records as the gold standard. A total of 34 patients with liver metastasis were recruited. The cut-off values of FR+CTC and HSP90 obtained from the ROC curve were used as boundary values to distinguish between high- and low-expression queues (cut-off value of FR+CTC=11.75, cut-off value of HSP90 = 55.65). According to the survival analysis of a Logrank test, the mDFS of a FR+CTC high-expression cohort is 36.5 months (95% CI: 14.13–58.87). Disease-free survival was completely different between high- and low-expression FR+CTC groups. Patients showing high expression of FR+CTC were more likely to develop liver metastasis than patients with low expression (Logrank *P* < 0.0001; **Figure 3C**). We also obtained a similar effect with HSP90. At 58.47 months, the mDFS of the HSP90 high-expression cohort was significantly higher than that of the low-expression cohort (Logrank *P* ;= 0.0002; 95% CI: 37.12–79.81; **Figure 3D**). Hence, from the detection efficiency of the two markers in the survival curve, the high expression of FR+CTC and HSP90 in serum is not conducive to the prognosis of patients; this significantly affects the tumor-free survival of patients. In addition, FR+CTC has a greater impact on the disease progression of liver metastasis in patients with colorectal cancer than HSP90. Thus, in some patients with colorectal cancer, the high expression of unilateral FR+CTC and HSP90 will significantly affect the survival time from initial treatment to liver metastasis. Whether FR+CTC and HSP90 can jointly affect survival need further exploration.

### Univariate and multivariate regression models of FR+CTC and HSP90 can predict the risk of liver metastasis in patients with colorectal cancer

**Figure 4:**
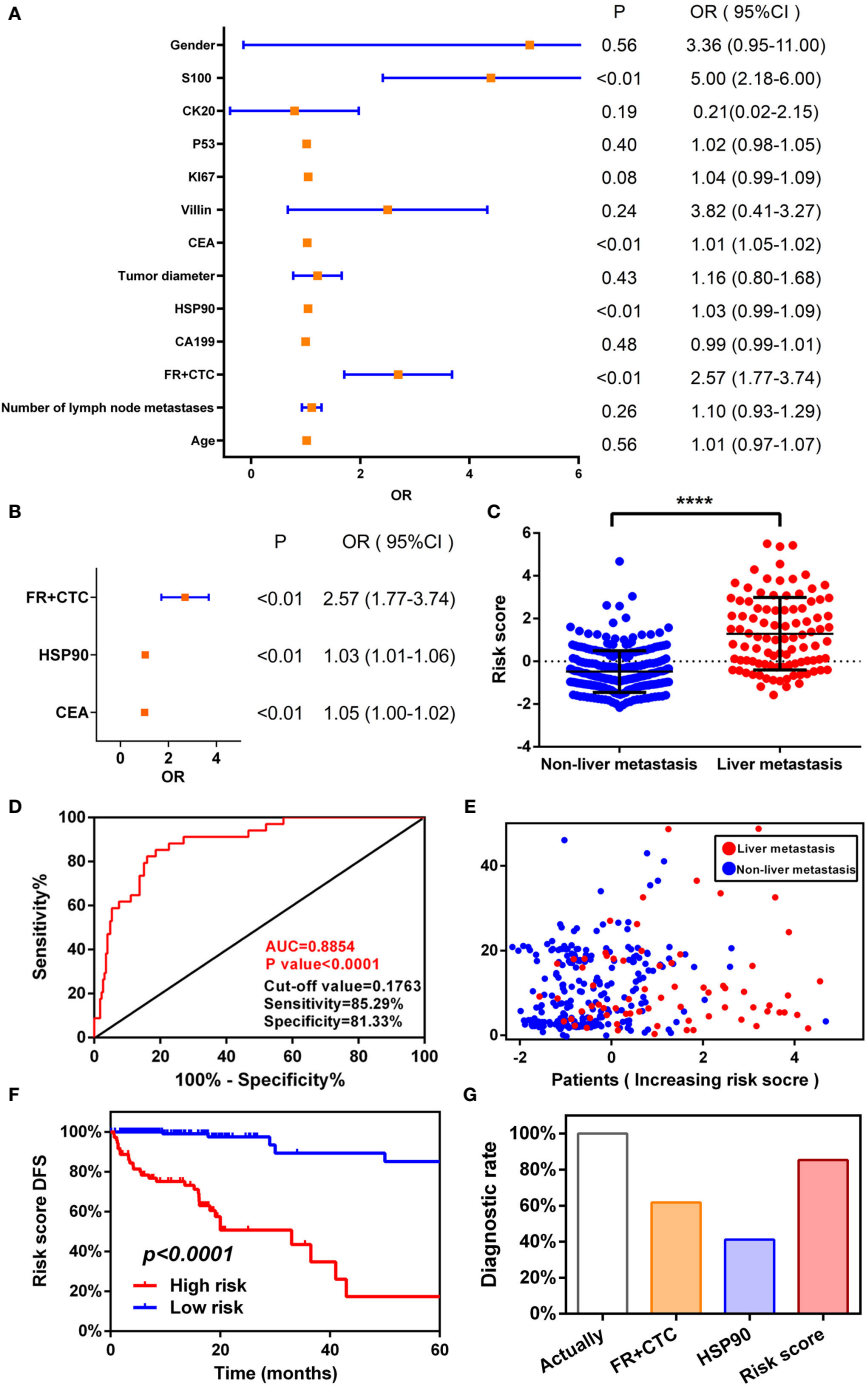
Establishing a logistic regression risk assessment model. Univariate **(A)** and multivariate **(B)** analyses of risk factors for liver metastasis of colorectal cancer. **(C)** Receiver operating characteristic (ROC) curve of risk scoring model. **(D)** Risk score model and survival scatter plot of patients with colorectal cancer. High risk of folate receptor plus circulating tumor cells (FR+CTC) **(C)** and heat shock protein 90 (HSP90) **(D)** predicts the median disease-free survival (mDFS) of liver metastatic colorectal cancer. **(E)** High- and low-risk scores predict mDFS in patients with colorectal cancer liver metastases. **(F)** Comparison of diagnostic ability between risk model and single index.

We followed up the previous non-metastatic cohort. We determined that 34 people reached the end point of the study (liver metastasis), and were divided into liver and non-liver metastasis groups. Univariate analysis of DFS showed that FR+CTC (*P* < 0.0001), HSP90 (*P* < 0.001), CEA (*P* < 0.0001), and S100 (*P* = 0.03) were more correlated with liver metastasis in patients with colorectal cancer (**Figure 4A**). In addition, the risk factors with statistical significance in the above univariate analysis were further included in the follow-up multivariate analysis. An appropriate Cox regression multivariate analysis model was established that showed that FR+CTC (*P* < 0.0001), HSP90 (*P* < 0.001), and CEA (*P* < 0.0001) were independent factors. FR+CTC, HSP90, and CEA were strong risk factors for liver metastasis in our study cohort (**Figure 4B**).

According to a multivariate Cox proportional hazards regression model, we obtained the expression coefficient of each independent risk gene. Taking into account these three risk factors, we established a prediction model of DFS to anticipate the risk of liver metastasis. After standardization of three indexes with a zero-centered method, the formula of the model was as follows: Risk score=(0.945×Standardized value of FR+CTC)+(0.034×Standardized value of HSP90)+(0.01×Standardized value of CEA). Then, we calculated the risk score of each patient. There was a significant difference in risk scores between the liver metastasis cohort and the non-liver metastasis cohort (*P* < 0.0001, Wilcoxon test)(**Figure 4C**). A ROC diagnostic test was performed on the risk score of the two groups (**Figure 4D**). We found that AUC=0.89 (*P* < 0.0001, 95% CI: 0.83–0.94), cut off=0.018, sensitivity=85.29%, and specificity=81.33%. A multivariate regression model was utilized to diagnose liver metastasis of colorectal cancer that is able to comprehensively consider the differences in individual markers of patients and predict the prognosis according to standardized score management. The diagnostic sensitivity was also greatly improved, which markedly enhanced diagnostic efficiency.

Next, using the cut-off value calculated from the ROC curve as the dividing point, the patients were divided into a high- (n=188) or low-risk groups (n=71). Combined with the disease progression (liver metastasis) time of each patient, a patient’s risk score time scatter diagram (**Figure 4E**) was generated. The higher the risk score of patients, the more patients with liver metastasis and the fewer patients without liver metastasis, resulting in the time of liver metastasis moving forward. Consistent with the predicted trend of the model, the model greatly screened out patients with high-risk liver metastasis.

We also performed a Kaplan–Meier curve for these four labeled risk factors according to the risk score (**Figure 4F**). A significant difference in survival rate was observed between high- and low-risk groups (Logrank *P* < 0.0001). The mDFS of the high-risk group was 33 months (95% CI: 10.16–44.88). The mDFS of the high-risk cohort was shorter than that of the low-risk cohort. Compared with the aforementioned single factor survival curve with respect to FR+CTC and HSP90, multiple factors consider the patient’s markers to predict recurrence and mDFS is shorter than that before, which greatly improves the efficiency of diagnosing liver metastasis and better predicts the time of liver metastasis.

Finally, from the follow-up data of 259 patients with non-liver metastatic colorectal cancer, 34 actually reached the end point of the study (liver metastasis). We used the cut-off values obtained from the ROC curves for FR+CTC and HSP90, as well as a risk score model, to count the number of patients with liver metastases. The use of FR+CTC eventually identified 21 patients with non-metastatic colorectal cancer who developed liver metastasis after initial treatment. The diagnosis rate was 61.76% (Diagnosis rate=Number of patients diagnosed by FR+CTC/Number of patients actually diagnosed). The use of HSP90 identified 14 patients with liver metastasis, and the diagnostic rate was 41.18%. The diagnostic rate of the risk score model was significantly higher than that of a single index (**Figure 4G**); it identified 29 people with liver metastasis, with a diagnostic rate of 85.29%. The risk score model greatly improved the positive rate of diagnosis and clinical practicability. Therefore, a combination of FR+CTC and HSP90 can improve diagnostic efficiency, lead to a better understanding of the prognosis of patients with colorectal cancer, allow timely clinical intervention, and improve the survival rate.

## Discussion

Liver metastasis with colorectal cancer is an important factor that seriously threatens the survival and prognosis of patients. Liver metastasis in patients is often asymptomatic and difficult to diagnose in the early stage. It takes time for metastases to be determined by imaging examinations (enhanced CT or PECT). Negative and misdiagnosed rates may exist even at an early stage (16). When multiple liver metastases are diagnosed by imaging, the opportunity for effective treatment has been lost, and the prognosis is quite poor. Consequently, we face the challenge of how to screen patients with a high risk of liver metastasis before signs of metastasis are found on imaging. Markers for this need to be relatively sensitive and specific, which will greatly promote treatment for colorectal cancer and improve the prognosis.

An analysis of our findings revealed that the combination of FR+CTC and HSP90 has a certain value in the diagnosis of colorectal cancer liver metastasis. A significant positive correlation was noted between FR+CTC and HSP90 in the liver metastasis cohort (*P* < 0.01). However, FR+CTC and HSP90 showed significant differences when compared with the non-liver metastasis cohort and were highly expressed in the metastasis cohort. In the non-metastatic cohort, the correlation between the two was not significant; in addition, the expression levels of FR+CTC and HSP90 were lower than those in the metastatic group. In addition, the numerical range was relatively small which was more stable and reduced the influence of individual differences and statistical errors in colorectal patients. We explored the diagnostic ability of FR+CTC and HSP90 to identify patients with liver metastases from colorectal cancer. This diagnostic ability was better than that of other blood markers. The sensitivity of HSP90 was higher than that of FR+CTC, but the specificity of FR+CTC was higher than that of HSP90, indicating a complementary diagnostic relationship to a certain extent. We also explored the ability of a FR+CTC and HSP90 combined diagnosis; AUC = 0.79, sensitivity was 70.55%, and specificity was 92.66%. What is notable is that a combined index showed a certain improvement in sensitivity and specificity compared with a single index in the diagnosis of liver metastases. This greatly improved the detection efficiency, which has a certain guiding significance for the early detection of colorectal cancer liver metastases.

We also compared our findings with commonly used tumor markers such as CEA and CA199. Although several differences occurred in the two cohorts, they are more commonly used to explain tumor tissue-specific expression associated with *in situ* colorectal cancer patients, since the mechanism of the direct link between cancer and liver metastases is still not fully demonstrated. It is impossible to screen for the occurrence of liver metastases at an early stage. However, tumor metastasis is closely connected to CTCs, which can directly reflect the metastasis of tumor cells in the blood. Circulating tumor cells can appear in the early stages of cancer metastasis. Such cells can enter the blood circulation through epithelial–mesenchymal transition (EMT), which enables tumor cells to transform the epithelial cell type and be able to penetrate the vessel wall, and are prone to causing distant metastases (17, 18). To date, more than 50 analytical methods have been applied to the identification of CTCs. The analytical methods are divided into two categories: one is based on cell surface markers, and the other is based on cell size and density. Both have many shortcomings that lead to their clinical applications not being widely utilized. Folate receptor α is the most widely studied isoform and shows restricted expression in normal cells. Folate receptor α expression is restricted to a few normal sites, including kidney, lung, choroid plexus, and placenta, but is highly expressed in various tumors of epithelial origin (19). In tumors, FRα is localized to the luminal surface of polarized epithelium and is highly expressed and stable in colorectal cancer epithelial cells. Our use of FR-labeled CTCs has a higher positive rate and sensitivity than the more commonly used CellSearch^®^ System EpCAM-labeled CTCs. The FR+CTC combination also shows high sensitivity and utility in the detection of lung, breast, hepatobiliary, and pancreatic cancers (20–23). In addition, FRα can promote the rapid growth and division of cells by regulating the uptake of folic acid in serum or by generating regulatory signals, which have a growth advantage for tumors. The involvement of FR in folate-induced JAK–STAT3 signaling is often activated in cancers of epithelial origin, promotes proliferation, and is associated with poor patient outcomes (24, 25); it also promotes signaling of the serine/threonine kinases, ERK1 and ERK2 (MAP kinase)/P53 in colorectal cancer tumor cells (26).

Combined with the high heterogeneity of colorectal cancer tumors and mutation analysis, we found that FR+CTC was significantly associated with HSP90. Previous studies have shown that HSP90 is also highly expressed in patients with colorectal cancer liver metastases, and is highly conserved and ubiquitously expressed in cancer tissues (27). Heat shock protein 90 can participate in the proliferation and invasion of cancer cells, and directly participates in the metastatic mechanism of tumors as a molecular chaperone. Heat shock protein 90 is equally currently considered a prospective therapeutic target for the treatment of oncoprotein-driven cancers. Recent studies have shown that HSP90 can promote the metastasis of colorectal cancer cells by regulating LASP1 abundance in a PUS7-dependent manner (28). Research shows Heat shock protein 90 promotes epithelial to mesenchymal transition, invasion, and migration in colorectal cancer (29). Both FR+CTC and HSP90 jointly participate in the process of EMT to a certain extent, so this combination from a liquid biopsy is more convincing in the diagnosis of patients with metastatic colorectal cancer. Therefore, we used the combined markers of FR+CTC and HSP90 to further explore the diagnostic efficacy and prognosis of colorectal cancer liver metastases. We also strove to detect high-risk colorectal cancer patients at an early stage, and to take timely intervention measures for an accurate diagnosis and treatment. Ultimately, this had the effect of improving the survival rate of patients.

We undertook a long-term follow-up study in a non-liver metastases cohort; the end point was the occurrence of liver metastases. We found consistent survival prognostic trends in high-expressing FR+CTC and HSP90 cohorts, respectively. The mDFS of a high-expressing FR+CTC patient cohort was 36.5 months, and the mDFS of a high-expressing HSP90 patient cohort was 58.47 months. The mDFS of the two lower expression groups was significantly shortened. Previous studies on the use of immunohistochemistry to detect FR in patients have also shown that it is not only associated with a five-year survival prognosis in colorectal cancer, but may also be associated with microsatellite instability status; it can also be used as an independent impact factor for patients with stage IV liver metastases (7). Activation of FRs is associated with survival and prognosis in cancer patients. In addition, high expression of HSP90 is associated with a poor prognosis in patients with colorectal cancer; our results are in agreement with those of previous studies (27). We believe that in patients with colorectal cancer under liquid biopsy, the high expression of FR+CTC and HSP90 shows a certain correlation with the occurrence of liver metastases, and has a relatively poor prognosis, which significantly affects the time of disease-free survival of patients. We also performed univariate analysis of DFS to explore influencing factors. We found that FR+CTC, HSP90, CEA, and S100 were all highly correlated with liver metastasis in patients with colorectal cancer. After adjusting for clinically significant univariate factors, multivariate logistic analysis showed that FR+CTC, HSP90, and CEA contributed to the model for patients with colorectal cancer liver metastases. These three markers can be considered as risk factors in the model.

The above studies demonstrate that metastatic colorectal cancer to the liver shows the characteristics of high expression of FR and HSP90, and is an influencing factor for the progression and prognosis of colorectal cancer. More recently, the research and development of FR and HSP90 inhibitors as adjuvant chemotherapy methods are being used in the management of clinical decisions on treatment. Investigations in FR approaches involving small molecules, folate drug conjugates, monoclonal antibodies, and vaccines are well under way. Farletuzumab can bind to FRα-mediated cytotoxicity and complement to promote cell death, and sustained tumor autophagy leads to impaired cell proliferation. Folic acid conjugates have been proposed as a promising strategy for the treatment of FRα-positive cancers (8). Folate–drug conjugates can lead to the release and diffusion of cytotoxic agents, and have achieved satisfactory results in studies of ovarian and lung cancers. In addition, HSP90 inhibitors are also being studied in patients with colorectal cancer: Phase I clinical trials of ganetespib, luminespib combined with capecitabine have achieved relatively optimistic results (30, 31). In addition, receptor tyrosine kinases (RTKs) and their signal transduction depend on the activity of HSP90 (15), EGFR, vascular endothelial growth factor (VEGF), and their corresponding RTK receptors. The receptor for VEGF (VEGFR) is a site of benefit for targeted therapy in patients with metastatic colorectal cancer (32). Studies of HSP90 inhibitors and anti-angiogenesis factors have shown great potential (33). A phase Ib trial of TAS-116 in colorectal cancer had the advantage of a known safety profile and antitumor activity, especially in patients with MSS colorectal cancer (34). This suggests that HSP90 inhibitors can be utilized to treat patients with advanced colorectal cancer. We propose a hypothesis that in patients with metastatic colorectal cancer with high expression of FR+CTC and HSP90, chemotherapy combined with targeting FRs and HSP90 inhibitors may have a certain therapeutic effect in future.

Finally, we calculated the expression coefficients of the three risk factors and normalized expression levels to obtain the cumulative risk score for each patient in the model. For the ROC curve, AUC = 0.89, sensitivity 85.29%, and specificity 81.33%. The sensitivity was considerably improved, indicating a further improvement in the screening efficiency of liver metastases. The risk score mDFS was 33 months, which was 3.5 months and 25.47 months earlier than FR+CTC and HSP90 on their own in predicting liver metastasis progression, respectively. By comparing the number of patients with liver metastases diagnosed by risk score, FR+CTC, and HSP90 with the actual number of patients diagnosed with liver metastases, the risk score group diagnosed more colorectal cancer patients, which shows that combined multivariate analysis can be more effective. Early identification of patients with high-risk liver metastases and high expression of FR+CTC and HSP90 indicates a poor prognosis.

This study had many limitations. One is the small sample size of the study cohort and the small number of colorectal cancer patients with liver metastases. All patients with colorectal cancer in this study came from the same hospital, which may have resulted in selection errors. Additionally, colorectal cancer is a heterogeneous entity. The occurrence and progression of tumors are inextricably linked to multiple site mutations; we did not assess mutational status, so we could not assess the correlation between FR+CTC, HSP90, and these molecular alterations. Third, the effect of FR and HSP90 on colorectal cancer metastasis at both gene and protein levels needs to be further elucidated in order to provide more evidence to prove the mutual regulatory relationship between the two indices.

In conclusion, the retrospective analysis of this small-scale exploratory study shows that the combination of FR+CTC and HSP90 is feasible and reliable enough to be used in the screening of colorectal cancer patients with liver and non-liver metastases. The high expression of both is not conducive to the prognosis of colorectal cancer and increases the risk of liver metastases. More importantly, a multivariate risk score model constructed by FR+CTC and HSP90 can be used as an applicable reference method for patients with liver metastatic colorectal cancer. Therefore, further investigation of the clinical implications in larger population cohorts is warranted.

## Data availability statement

The raw data supporting the conclusions of this article will be made available by the authors, without undue reservation.

## Ethics statement

The studies involving human participants were reviewed and approved by Guangxi Medical University Cancer Hospital. The patients/participants provided their written informed consent to participate in this study. Written informed consent was obtained from the individual(s) for the publication of any potentially identifiable images or data included in this article.

## Author contributions

MH and LY designed the study, obtained clinical information, contributed to the design, experimental work and analysis. LC and HR obtained and documented clinical information. XM and SM recruited patients and obtained clinical information. LY contributed to experimental design and provided funding. All authors contributed to the article and approved the submitted version.

## Funding

This research was supported by The National Science Foundation (No.82160495). Guangxi University High-level Innovation Team and the Project of Outstanding Scholars Program (2019AC03004) and Guangxi Science and Technology Project (AD19245197). This work was supported by China Postdoctoral Science Foundation (No. 2019M653812XB).

## Conflict of interest

The authors declare that the research was conducted in the absence of any commercial or financial relationships that could be construed as a potential conflict of interest.

## Publisher’s note

All claims expressed in this article are solely those of the authors and do not necessarily represent those of their affiliated organizations, or those of the publisher, the editors and the reviewers. Any product that may be evaluated in this article, or claim that may be made by its manufacturer, is not guaranteed or endorsed by the publisher.

## Supplementary material

Supplementary Figure 1 Colorectal cancer patients with liver metastasis (CT or PECT).
