# Supplementary figures and images for: Evaluation of colorectal cancer liver metastases based on liquid biopsy combined with folate receptor– Positive circulating tumor cells and HSP90
Source: Front Oncol. 2022 Sep 20;12:912016. doi: 10.3389/fonc.2022.912016 (PMC9531159; doi:10.3389/fonc.2022.912016)

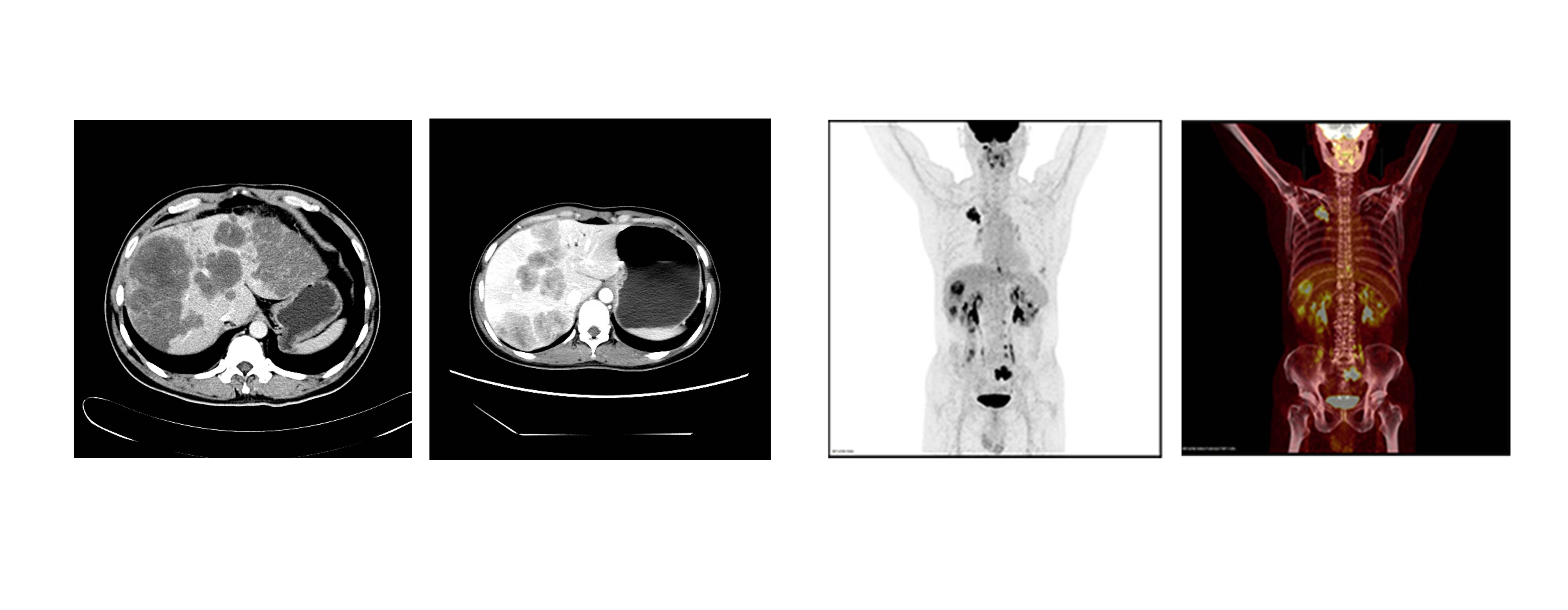

Supplement: Supplementary Figure 1 — Colorectal cancer patients with liver metastasis (CT or PECT). [file Image_1.tif]
